# Hypermethylation suppresses microRNA-219a-2 to activate the ALDH1L2/GSH/PAI-1 pathway for fibronectin degradation in renal fibrosis

**DOI:** 10.21203/rs.3.rs-2986934/v1

**Published:** 2023-06-05

**Authors:** Xiao Xiao, Emily Huo, Chunyuan Guo, Xiangjun Zhou, Xiaoru Hu, Charles Dong, Huidong Shi, Zheng Dong, Qingqing Wei

**Affiliations:** Zhongnan Hospital of Wuhan University; Augusta Preparatory Day School; Shanghai Skin Disease Hospital, Tongji University School of Medicine; Renmin Hospital of Wuhan University; The Second Xiangya Hospital at Central South University; Augusta University; Augusta University; Augusta University; Augusta University/Medical College of Georgia

## Abstract

Epigenetic regulations, such as DNA methylation and microRNAs, play an important role in renal fibrosis. Here, we report the regulation of microRNA-219a-2 (mir-219a-2) by DNA methylation in fibrotic kidneys, unveiling the crosstalk between these epigenetic mechanisms. Through genome-wide DNA methylation analysis and pyro-sequencing, we detected the hypermethylation of mir-219a-2 in renal fibrosis induced by unilateral ureter obstruction (UUO) or renal ischemia/reperfusion, which was accompanied by a significant decrease in mir-219a-5p expression. Functionally, overexpression of mir-219a-2 enhanced fibronectin induction during hypoxia or TGF-β1 treatment of cultured renal cells. In mice, inhibition of mir-219a-5p suppressed fibronectin accumulation in UUO kidneys. ALDH1L2 was identified to be the direct target gene of mir-219a-5p in renal fibrosis. Mir-219a-5p suppressed ALDH1L2 expression in cultured renal cells, while inhibition of mir-219a-5p prevented the decrease of ALDH1L2 in UUO kidneys. Knockdown of ALDH1L2 enhanced PAI-1 induction during TGF-β1 treatment of renal cells, which was associated with fibronectin expression. In conclusion, the hypermethylation of mir-219a-2 in response to fibrotic stress attenuates mir-219a-5p expression and induces the up-regulation of its target gene ALDH1L2, which may reduce fibronectin deposition by suppressing PAI-1.

## Introduction

Chronic kidney disease (CKD) is a highly prevalent renal disease that affects about 15% of adults in the USA (https://nccd.cdc.gov/CKD/Default.aspx). CKD is featured by a gradual loss of renal function with various risk factors including hypertension, diabetes, autoimmune disorders, and acute kidney injury transition to CKD. Renal fibrosis, a pathological hallmark of CKD, is characterized by excessive accumulation of extracellular matrix (ECM) proteins including fibronectin and collagens. Currently, effective therapy which can reverse or delay renal fibrosis is lacking. Various signaling pathways, including TGF-β signaling pathway and HIF pathway, play pivotal roles in renal fibrosis by promoting ECM protein production^1–3^. The ECM is continuously undergoing degradation and remodeling. The imbalance between ECM protein expression and degradation leads to excessive ECM deposition and flbrosis^4^. However, the understanding of dysregulation of ECM remodeling in renal fibrosis remains incomplete.

Epigenetic regulation includes the mechanisms that modulate gene expression inheritably without changing the primary DNA sequence. DNA methylation is one of the epigenetic mechanisms that broadly affect gene expression by adding a methyl group to the cytosine at a CpG site through a reaction catalyzed by DNA methyltransferases^5^. Functionally, the methylation of CpG sites in the promotor region of a gene usually suppresses the transcription of the gene. Aberrant DNA methylation has been implicated in various diseases, including CKD^6–13^. However, it remains poorly understood how DNA methylation contributes to the development and progression of CKD.

Non-coding RNAs, including microRNAs, represent another major mechanism of epigenetic regulation. MicroRNAs are a group of small non-coding RNAs of 21–25 nucleotides. By binding to the target sequence in the 3’-untranslated region (UTR) of mRNAs, microRNAs can suppress gene expression by inhibiting mRNA translation and/or inducing mRNA degradation. Multiple microRNAs, such as microRNA-21, microRNA-192, microRNA-214, and microRNA-29, have been shown to play important roles in CKD and renal fibrosis^14–19^. MicroRNA-219a (mir-219a) is encoded by two separate genes (mir-219a-1 and mir-219a-2), but both genes produce the same leading strand mir-219a-5p. Mir-219a-5p has been reported to regulate neurodegeneration, nerve system development, and cancer progression^20–23^. There is no report about mir-219a in kidney diseases including CKD and renal fibrosis. Furthermore, the mechanism that controls mir-219a gene expression is unclear.

In this study, we examined DNA methylation changes in mouse models of renal ischemia/reperfusion and unilateral ureter obstruction (UUO). Our genome-wide DNA methylation analysis identified the hypermethylation of mir-219a-2 at its gene promotor region in these models, which was associated with a decrease in mir-219a-5p expression. Functionally, mir-219a-5p was shown to directly target and repress ALDH1L2, leading to the suppression of reduced glutathione (GSH) production, induction of plasminogen activator inhibitor 1 (PAI-1), and accumulation of fibronectin. These findings unveil hypermethylation of mir-219a-2 as a novel anti-fibrotic mechanism that is activated in renal tubular cells in renal fibrosis.

## Results

### Hypermethylation of Mir-219a-2 is accompanied by decreased mir-219a-5p expression in fibrotic kidneys.

**Figure 1:**
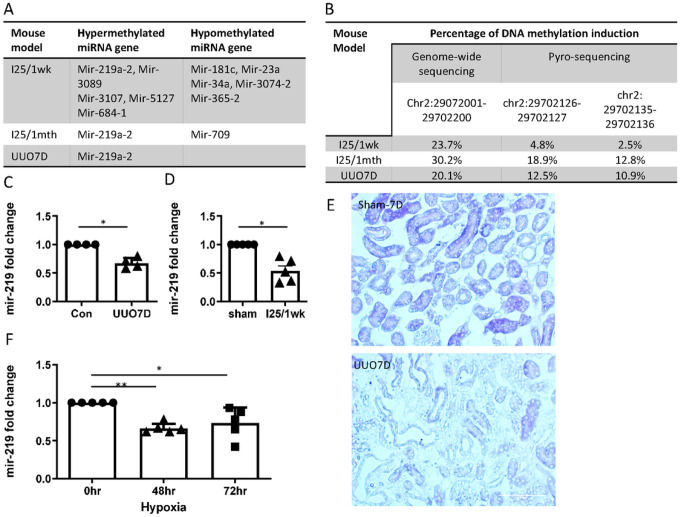
Hypermethylation of Mir-219a-2 is accompanied by decreased mir-219a-5p expression in fibrotic kidneys. (A) & (B) DNA methylation level in C57BL/6J mouse kidneys after 25 minutes of bilateral ischemia with 1-week reperfusion (I25/1wk), or 25 minutes of bilateral ischemia with 1 month reperfusion (I25/1mth), or unilateral ureteral obstruction 7 days (UUO7D) was compared to sham control kidneys. Pooled DNA samples from 3 mice/group (I25/1wk, I25/1mth, vs sham) or 2 mice/group(UUO7D vs control) were used for genome-wide DNA methylation analysis. (A) The list of differentially methylated microRNA genes. The microRNA genes were identified with >20% methylation level difference in the promotor region comparing to control or sham. (B) The methylation level of mir-219a-2 gene was examined by genome-wide DNA methylation sequencing and pyro-sequencing. The percentage of DNA methylation induction was calculated comparing to sham or control. (C) qPCR analysis of mir-219a-5p in control and UUO7D mouse kidneys. *, P=0.0058, t=7.099, df=3, n=4, paired t-test. (D) qPCR analysis of mir-219a-5p in sham control and I30/1wk mouse kidneys. *, P=0.0070, t=5.102, df=4, n=5, paired t-test. (E) In situ hybridization of mir-219a-5p in sham control (Sham-7D) or 7 days of UUO mouse kidneys (UUO7D). Scale bar, 0.1mm. Representative images from two experiments. (F) mir-219a-5p in control BUMPT cells or cells treated with hypoxia (1% O2) for 48 hours and 72 hours. One-way ANOVA (F=10.05) with uncorrected Fisher’s LSD for multiple comparisons. *, P=0.0051, t=3.413, DF=12; **, P=0.0009, t=4.356, DF=12.

We analyzed genome-wide DNA methylation changes by reduced representation bisulfite sequencing in models with significant renal fibrosis (Supplementary Fig. 1), including 25 minutes of bilateral renal ischemia followed by 1-week reperfusion (I25/1wk) or 1 month reperfusion (I25/1M), and unilateral ureter obstruction for 7 days (UUO7D). Interestingly, in addition to protein-coding genes (data not shown), we identified multiple microRNA genes with differential methylation in fibrotic kidneys compared to control kidneys (Fig. 1A). Among them, mir-219a-2 was the only one showing significant hypermethylation (> 20%) (mean methylation values of 5 CpG sites) at the 5’-end promotor region of the gene in all models tested (Fig. 1A, 1B). We further confirmed the hypermethylation of two specific CpG sites in the mir-219a-2 promotor region in these kidneys by pyrosequencing (Fig. 1B).

Mir-219a is encoded by two genes, i.e. mir-219a-1 and mir-219a-2, leading to the expression of the same highly conserved leading strand mir-219a-5p in various species including human, mouse, and rat (Supplementary Fig. 2). We speculated that hypermethylation at mir-219a-2 promotor region would attenuate mir-219a-5p expression in renal fibrosis. Indeed, qPCR analysis showed that mir-219a-5p significantly decreased in fibrotic kidneys after UUO7D and I25/1wk (Fig. 1C and 1D). In situ hybridization further verified the decrease of mir-219a-5p in renal tubular cells after UUO (Fig. 1E). In vitro, hypoxia for 48 hours and 72 hours significantly inhibited mir-219a-5p expression in mouse proximal tubular cells (BUMPT) (Fig. 1F). In addition, mir-219a-5p was significantly reduced in human kidney tubules with obstructive nephropathy (Supplementary Fig. 3). Collectively, these results demonstrate the hypermethylation of mir-219a-2 in fibrotic stress that is accompanied by the suppression of mir-219a-5p expression.

### Mir-219a-2 enhances fibronectin expression during hypoxia or TGF-β1 treatment of renal tubular cells.

**Figure 2:**
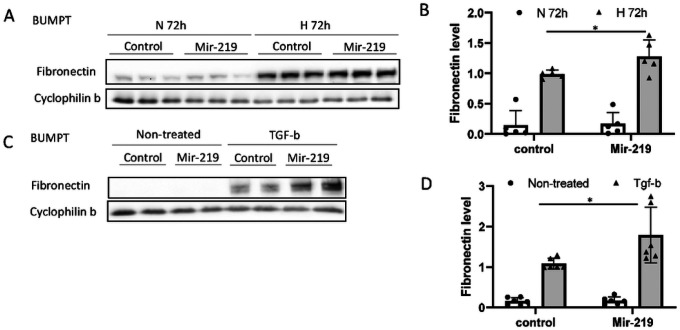
Mir-219a-2 enhances fibronectin expression during hypoxia or TGF-b1 treatment of renal tubular cells. (A) & (B) BUMPT cells with stable transfection of mir-219a-2 (mir-219) or empty vector (control) were treated with (H 72h) or without (N 72h) hypoxia (1% O_2_) for 72 hours to collect lysate for immunoblot analysis. (A) Representative immunoblots. (B) Densitometry analysis of fibronectin by normalization with cyclophilin b (loading control). N=5; *, Two-way ANOVA with uncorrected Fisher’s LSD for multiple comparisons, P=0.0436, t=2.190, DF=16. (C) & (D) BUMPT cells with stable transfection of mir-219a-2 (mir-219) or empty vector (control) were treated with or without 10ng/ml TGF-b1 for 72 hours to collect lysate for immunoblot analysis. (C) Representative images of immunoblots. (D) Densitometry analysis of fibronectin by normalization with cyclophilin b. N=6; *, Two-way ANOVA with uncorrected Fisher’s LSD for multiple comparisons, P=0.0027, t=3.414, DF=20.

Renal interstitial fibrosis involves the accumulation or deposition of multiple ECM components such as fibronectin and collagens in the interstitium space. To understand the role of mir-219a-5p in renal fibrosis, we established a mir-219a-2 gene overexpressing BUMPT cell line. mir-219a-2 overexpression in this cell line was confirmed by qPCR analysis (Supplementary Fig. 4). mir-219a-2 overexpressing cells and their control cells were treated with hypoxia (Fig. 2A, 2B) or TGF-β1 (Fig. 2C, 2D) to induce fibrotic changes, shown by significant accumulation of fibronectin in immunoblots. mir-219a-2 overexpressing cells showed significantly more fibronectin accumulation. Consistently, mir-219a-5p mimics induced fibronectin accumulation in rat renal proximal tubular cells (RPTCs) during hypoxia treatment (Supplementary Fig. 5A, 5B) and in human HK2 renal proximal tubular cells during TGF–β1 treatment (Supplementary Fig. 5C, 5D). Similar effects of mir–219a–5p mimics were shown in HEK293 (human embryonic kidney) cells (Supplementary Fig. 5E, 5F) during TGF–β1 treatment. These cell culture results suggest a pro-fibrotic role of mir-219a-5p in kidney.

### Antagonism of mir-219a-5p suppresses fibronectin accumulation in fibrotic kidneys.

**Figure 3:**
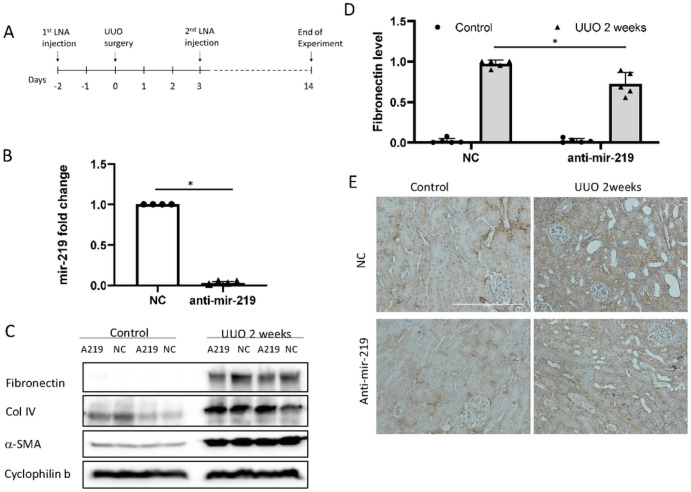
Antagonism of mir-219a-5p suppresses fibronectin expression in UUO-induced renal fibrosis. C57BL/6J male mice were treated with negative control (NC) or anti-mir-219a-5p (anti-mir-219 or A219) LNAs, and then subjected to unilateral ureteral obstruction (UUO) for 2 weeks. The whole lysates of the contralateral (control) and obstructive kidneys (UUO 2weeks) were examined by immunoblotting. (A) Schematic diagram showing the mouse treatment procedure. (B) qPCR analysis of mir-219a-5p in mouse kidneys. N=4, *, unpaired t-test, P<0.0001, t=108..8, DF=6. (C) Representative immunoblots of fibrotic proteins. (D) Densitometry analysis of fibronectin in mouse kidneys. The value was normalized by cyclophilin b. N=5, Two-way ANOVA with uncorrected Fisher’s LSD for multiple comparisons. *, P=0.0001, t=5, DF=16. (E) Immunohistochemical staining of fibronectin in control and UUO kidneys (UUO 2weeks). Scale bar=0.2mm.

To examine the role of mir-219a-5p in renal fibrosis in vivo, we used anti-mir-219a-5p LNAs to treat mice and then subjected them to UUO for 2 weeks (Fig. 3A). The inhibition of mir-219a-5p by LNA treatment in mouse kidneys was confirmed by qPCR (Fig. 3B). UUO induced significant tubulointerstitial fibronectin, shown by the increase of total protein expression in immunoblots (Fig. 3C, 3D) and immunohistochemical staining (Fig. 3E). Notably, anti-mir-219a-5p LNAs reduced the accumulation of interstitial fibronectin as shown by both immunoblots (Fig. 3C, 3D) and immunohistochemical staining (Fig. 3E).

Interestingly, anti-mir-219a-5p did not affect the expression of other renal fibrosis marker proteins, such as col IV and α-SMA (Fig. 3C, Supplementary Fig. 6C, 6D). Consistently, histological examinations did not show noticeable effects of anti–mir–219a–5p on α–SMA induction (Supplementary Fig. 6A) or overall collagen deposition in UUO kidneys (Supplementary Fig. 6B, 6E). Thus, mir–219a–5p may contribute to renal fibrosis mainly by regulating fibronectin expression.

### mir-219 targets ALDH1L2 in renal fibrosis.

**Figure 4:**
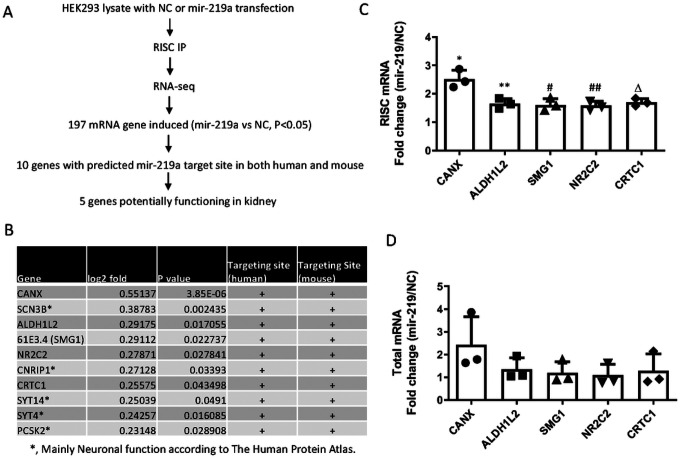
Identification of potential targets of mir-219a-5p. HEK293 cells were co-transfected with Flag-Ago2 plasmid and mir-219a-5p mimics or its negative control oligos (NC). RNA samples were extracted from whole cells (total mRNA) or Ago2 immunoprecipitates (RISC mRNA) for deep RNA sequencing (RNA-seq), n=3. (A) Schematics indicating the procedure to identify potential mir-219a-5p targets. (B) The list of potential targets with predicted mir-219a-5p binding sites in both human and mouse. (C) qPCR to confirm the mRNA levels of potential targets in RISC. P value (vs NC): *, P=0.0013, t=8.100, df=4; **, P=0.0030, t=6.414, df=4; #, P=0.0088, t=4.776, df=4; ##, P=0.0027, t=6.651, df=4; D, P=0.0008, t=9.178, df=4. (D) qPCR analysis of total RNA samples. The mRNA levels were compared between the cells transfected with mir-219a-5p mimics and those with negative control oligos.

**Figure 5:**
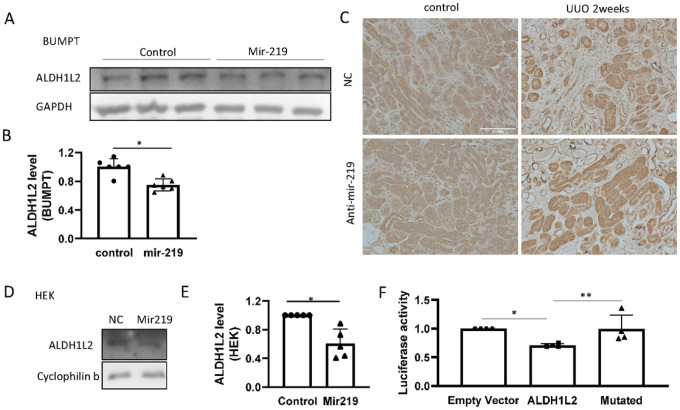
Confirmation of ALDH1L2 as a mir-219a-5p direct target. (A) Representative immunoblot of ALDH1L2 in BUMPT cells stably transfected with mir-219a-2 or empty vector (control). (B) Densitometry analysis of ALDH1L2 in BUMPT cells (normalized by GAPDH). *, student t-test, P=0.0013, t=4.423, df=10, n=6. (C) Immunohistochemical staining of ALDH1L2 in mouse kidney samples with negative control (NC) or anti-mir-219–5p LNA (Anti-mir-219) treatment (three repeats). Scale bar=0.1mm. (D) Immunoblots of ALDH1L2 in HEK293 cells transiently transfected with negative control (NC) or mir-219a mimics. (E) Densitometry analysis of ALDH1L2 in HEK293 cells normalized with cyclophilin b level. *, P=0.0024, t=4.361, df=8, n=5. (F) Luciferase assay to confirm the binding of mir-219a-5p to the predicted binding site in ALDH1L2 3’-UTR. HEK293 cells were co-transfected with RNA oligos (negative control or mir-219a-5p mimics) and luciferase plasmids without insertion (empty vector), or with insertion [predicted mir-219a-5p binding site of ALDH1L2 (ALDH1L2), or mutated binding site (mutated)] in the 3’-UTR of luciferase. The luciferase ratio between mir-219a-5p vs negative control was calculated for each group and then normalized by values from empty vector group. One-way ANOVA (F=5.474) with uncorrected Fisher’s LSD for multiple comparisons. N=4; *, P=0.0173, t=2.910, DF=9; **, P=0.0201, t=2.819, DF=9.

MicroRNAs repress target genes by binding to their mRNAs in the RNA-induced silencing complex (RISC) to suppress translation. In our experiments, mir-219a-5p enhanced fibronectin expression (Figs. 2, 3), indicating that fibronectin was not a direct target of mir-219a-5p. To elucidate the underlying mechanism, we systematically identified the direct targets of mir-219a-5p in renal cells (Fig. 4A). Specifically, we performed RISC-immunoprecipitation (RISC-IP) to pull down the potential target mRNAs of mir-219a-5p for deep sequencing, revealing 197 mRNAs (Supplementary Table 1). We then analyzed the potential mir-219a-5p targeting sites in the 3’-UTR of these mRNAs by an online database (http://www.microrna.org)^24^ and identified 10 mRNAs with conserved mir-219a-5p targeting sites in both human and mouse (Fig. 4B). We further searched the online database (The Human Protein Atlas, https://www.proteinatlas.org) to examine the protein expression and function of these 10 potential target genes^25^. Five of these genes mainly showed expression and function in neurons and were excluded from further study. For the other five genes (CANX, ALDH1L2, SMG1, NR2C2, CRTC1), we verified that mir-219a-5p induced significant accumulation of these 5 gene mRNAs in RISC, while it did not induce obvious changes in cellular levels of these mRNAs (Fig. 4C, 4D). Thus, we considered them as the potential direct targets of mir-219a-5p in renal cells. To further identify the target gene that is responsible for the effect of mir-219a-5p in renal fibrosis, we examined the protein expression of these potential targets. Mir-219a-5p did not change CANX protein expression, although it induced the highest mRNA accumulation of CANX in RISC (Supplementary Fig. 7). The second most accumulated mRNA induced by mir-219a-5p in RISC was ALDH1L2 (Fig. 4B). In BUMPT cells, mir-219a-2 overexpression significantly suppressed ALDH1L2 protein expression (Fig. 5A & 5B). Similarly, in HEK293 cells, mir-219a-5p mimics inhibited ALDH1L2 expression (Fig. 5D, 5E). In mouse kidneys, ALDH1L2 was mainly localized in renal tubular cells (Fig. 5C). Renal fibrosis in UUO was associated with a remarkable loss of ALDH1L2 in a large portion of renal tubules, which was prevented by anti-mir-219a-5p (Fig. 5C, supplementary Fig. 8). In luciferase microRNA target assay, the luciferase expression was significantly suppressed by mir-219a-5p in the presence of the predicted binding sequence at 3’-UTR, whereas it had no inhibitory effect when the binding sequence was mutated (Fig. 5F, Supplementary Fig. 9). Together, these data indicate that ALDH1L2 is a direct target of mir-219a-5p in renal fibrosis.

### ALDH1L2 promotes GSH accumulation and inhibits PAI-1.

**Figure 6:**
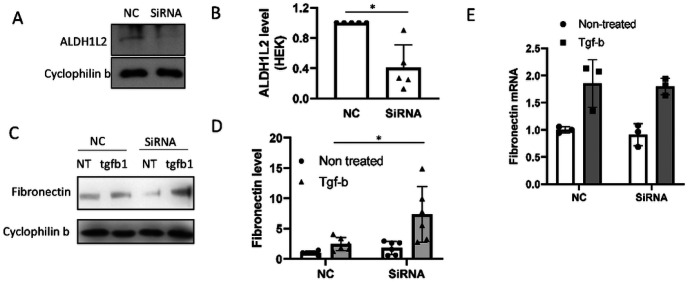
ALDH1L2 suppresses fibronectin protein induction. HEK293 cells were transfected with ALDH1L2 siRNA (SiRNA) or negative control oligos (NC). (A) Immunoblot analysis verifying ALDH1L2 knockdown in siRNA transfected cells. (B) Densitometry analysis of ALDH1L2 normalized by cyclophilin b. *, unpaired t-test, P=0.0024, t=4.358, df=8. (C) - (E) ALDH1L2 siRNA or negative control oligo-transfected HEK293 cells were treated with 20ng/ml TGF-b1 for 24 hours. (C) Representative immunoblot of fibronectin. (D) Densitometry analysis of fibronectin normalized by cyclophilin b or b-actin. Two-way ANOVA with uncorrected Fisher’s LSD for multiple comparisons. N=6; *, P=0.0021, t=3.530, DF=20. (E) qPCR analysis of fibronectin mRNA. N=3.

**Figure 7:**
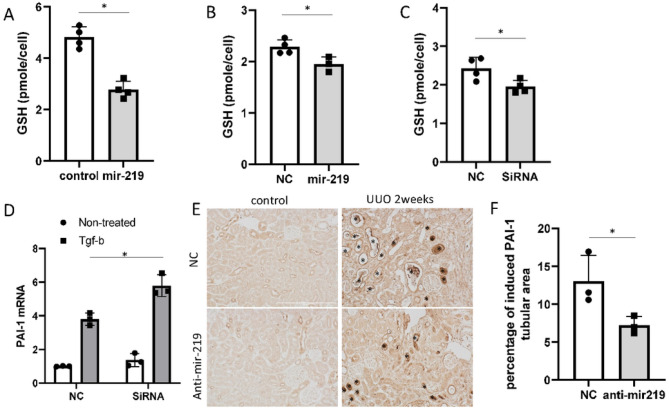
ALDH1L2 promotes GSH accumulation and inhibits PAI-1. (A) GSH concentrations in BUMPT cells stably transfected with mir-219–2 or empty vector (control). *, unpaired t-test, P=0.0003, t=7.681, df=5.754, n=4. (B) GSH concentrations in HEK293 cells with negative control (NC) or mir-219a-5p mimic transfection. *, unpaired t-test, P=0.0334, t=3.079, df=4.311, n=4. (C) GSH concentrations in HEK293 cells with negative control (NC) or ALDH1L2 siRNA transfection. *, unpaired t-test, P=0.0351, t=2.889, df=4.895, n=4. (D) HEK293 cells were transfected with ALDH1L2 siRNA (SiRNA) or negative control sequence (NC). PAI-1 mRNA was examined by qPCR. Two-way ANOVA with uncorrected Fisher’s LSD for multiple comparisons, N=3. P=0.004, t=5.805, DF=8. (E) & (F) Mice were treated with negative control (NC) or anti-mir-219a-5p LNA and subjected to UUO for 2 weeks. (E) Immunohistochemical images of PAI-1 in mouse kidney samples with or without UUO. Scale bar=0.2mm. *, renal tubules with significant induction of PAI-1. (F) Percentage of the renal tubular area with significant PAI-1 induction after 2 weeks of UUO. Unpaired t-test, P=0.0498, t=2.781, df=4, n=3.

**Figure 8:**
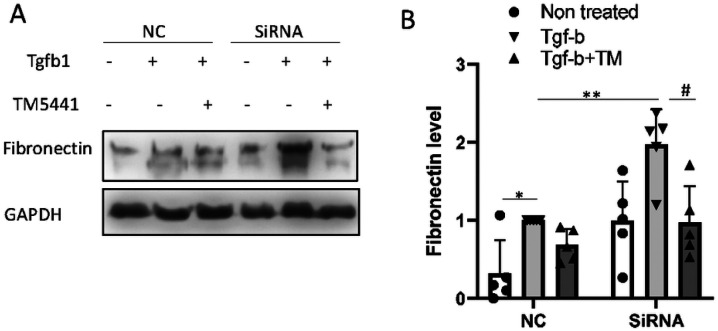
Inhibition of PAI-1 with TM5441 prevents fibronectin induction during TGF-b1 treatment. (A), (B): HEK293 cells with NC or ALDH1L2 siRNA were treated with PAI-1 inhibitor TM5441 and then subjected to TGF-b1 treatment. (A) Representative immunoblots of fibronectin with GAPDH as the loading control. (B) Densitometry analysis of fibronectin normalized with GAPDH or cyclophilin b signal. Two-way ANOVA with uncorrected Fisher’s LSD for multiple comparisons, N=5. *, P=0.0109, t=2.760, df=24; **, P=0.0006, t=3.938, df=24; #, P=0.0005, t=4.038, df=24.

To determine the role of ALDH1L2 in regulating fibronectin expression, we knocked down ALDH1L2 with specific siRNAs in HEK293 cells (Fig. 6A, 6B). ALDH1L2 knockdown cells had higher levels of fibronectin expression during TGF-β1 treatment than negative control (NC) sequence-transfected cells (Fig. 6C, 6D), indicating an anti-fibrotic role of ALDH1L2. Interestingly, ALDH1L2 knockdown increased fibronectin protein without changing fibronectin mRNA expression (Fig. 6E), suggesting ALDH1L2 regulates fibronectin at the level of protein turnover. ALDH1L2 is a mitochondrial folate metabolic enzyme that mediates fatty acid metabolism, and its depletion leads to impaired production of GSH and enhanced oxidative stress^26,27^. Reduction of GSH can up-regulate PAI-1 to suppress plasmin-mediated fibronectin degradation^28^. Therefore, we examined the possible connections between mir-219a-5p, ALDH1L2, and Fibronectin. Mir-219a-2 overexpression or mir-219a-5p mimics led to significant decreases in GSH in BUMPT cells and HEK293 cells, respectively (Fig. 7A, 7B). Similarly, the knockdown of ALDH1L2 decreased GSH in HEK293 cells (Fig. 7C). TGF-β1 induced PAI-1 as reported previously^28^, and this induction was markedly higher in ALDH1L2 knockdown cells (Fig. 7D). In vivo, UUO induced PAI-1 in specific renal tubules, which was suppressed by anti-mir-219a-5p (Fig. 7E, 7F). To confirm the role of PAI-1 in renal fibrosis, we tested the effect of TM5441, a specific PAI-1 inhibitor. As shown in Fig. 8, TM5441 reduced fibronectin expression during TGF-β1 treatment in ALDH1L2 knockdown cells to the level of negative control siRNA-transfected cells. Collectively, these results indicate that mir-219a-5p may repress ALDH1L2 to induce PAI-1 resulting in fibronectin accumulation.

## Discussion

In this study, we deciphered a novel crosstalk between two epigenetic mechanisms, DNA methylation and microRNA, in renal fibrosis. The genome-wide DNA methylation sequencing revealed mir-219a-2 as the only hypermethylated microRNA gene in mouse models with renal fibrosis, including ureter obstruction and maladaptive repair after renal ischemia-reperfusion injury (Fig. 1A, 1B). Furthermore, this mir-219a-2 hypermethylation is associated with the suppression of mir-219a-5p expression (Fig. 1C–1F) both in vivo and in vitro. All these results indicate the importance of mir-219a-2 hypermethylation in renal fibrosis. Although various aberrant epigenetic regulations, including both DNA methylation and microRNA regulation, have been noted as critical pathological mechanisms, the crosstalk between different epigenetic mechanisms is not well understood. DNA methylation has been reported to regulate microRNA biogenesis^29^ and DNA methylation-related microRNA expression change has been profiled in diabetic nephropathy^30^. However, a detailed functional analysis of such crosstalk is lacking. Notably, our study elucidated a crosstalk between DNA methylation and microRNA regulation by comprehensive examinations of the mir-219a-2 promotor hypermethylation, mir-219a-5p inhibition, and the downstream protein factors regulated by mir-219a-5p in renal fibrosis.

Moreover, the new pro-fibrotic function of mir-219a-5p was identified in renal fibrosis. We examined BUMPT cells with mir-219a-2 overexpression and identified that this overexpression led to more fibronectin expression in vitro (Fig. 2). The pro-fibrotic effect of mir-219a-5p was further confirmed in a few other renal cell lines including rat proximal tubular cells (RPTC), human proximal tubular cells (HK2), and HEK293 cells (Supplementary Fig. 5). In vivo, the inhibition of mir-219a-5p was mainly localized in renal tubules in UUO kidneys (Fig. 1E). If mice were treated with anti-mir-219a-5p LNAs, the interstitial fibronectin accumulation in the kidneys after UUO injury was relieved (Fig. 3). Meanwhile, we did not detect an obvious effect on α-SMA (the fibroblast marker). Although renal fibroblast is one of the major contributors to ECM accumulation in renal fibrosis, emerging evidence indicates that renal tubular cells are playing critical roles in renal fibrosis initiation and progression^31^. All these data indicate that the suppression of mir-219a-2 gene expression by DNA hypermethylation may act as a self-protection mechanism for the kidney to prevent fibrosis development in CKD, and renal tubular cells are the major functional sites of mir-219a-5p.

The function of mir-219a-5p has been examined previously in neurons and cancer cells^20–23^. In those studies, multiple pathways were reported to be suppressed by mir-219a-5p, including the NMDA receptor pathway, Tau, Hedgehog signaling pathway, and Wnt/β-catenin pathway Therefore, mir-219a-5p may target different genes in different tissues or organs. To identify the target genes in renal fibrosis, we performed Ago2-IP with deep sequencing, followed by the examination of putative target protein expression and the luciferase microRNA target assay. These analyses identified ALDH1L2 as a direct target of mir-219a-5p in renal fibrosis, especially fibronectin expression (Figs. 4 & 5). ALDH1L2 is a mitochondrial homolog of 10-formyltetrahydrofolate dehydrogenase, which controls the β-oxidation in fatty acid metabolism^26,27^. There is no report about ALDH1L2 in renal pathophysiology. Fatty acid oxidation is a major energy source in kidney proximal tubular cells, and its dysregulation is closely involved in renal fibrosis^32–34^. In our experiment, the knockdown of ALDH1L2 significantly enhanced fibronectin expression during TGF-β1 treatment (Fig. 6A–6D), suggesting that ALDH1L2 is a key downstream target of mir-219a-5p to regulate renal fibrosis. In vivo, anti-mir-219a-5p prevented the decrease of ALDH1L2 in UUO injured kidneys, which was associated with less fibronectin induction (Fig. 5C). Altogether, these results demonstrate the important role of ALDH1L2 as a mir-219a-5p target in renal fibrosis.

How does ALDH1L2 suppression lead to renal fibrosis? In HEK293 cells, ALDH1L2 knockdown increased fibronectin protein but not its mRNA (Fig. 6), suggesting the regulation of fibronectin protein stability by ALDH1L2. Fibronectin is one of the major components of ECM that contributes to flbrosis^4^. Especially, fibronectin polymerization is an essential step for other ECM proteins to deposit in the fibrotic niche^35^. The degradation of fibronectin and related ECM remodeling involves two major families of metalloproteinases, matrix metalloproteinases (MMPs) and plasmin^4^. MMPs degrade a variety of ECM proteins, whereas plasmin is more specific for degrading fibronectin, fibrin, and laminin to regulate ECM remodeling. Our results showed that PAI-1 expression was induced after ALDH1L2 knockdown (Fig. 7D). PAI-1 is the principal plasminogen activator inhibitor to promote plasminogen truncation to be plasmin^36^, and its expression can be regulated by GSH level^28^. In this regard, ALDH1L2 promotes the production of GSH in cells^27^, which may suppress PAI-1 expression and induce plasminogen to plasmin conversion for fibronectin degradation^28^. Consistently, in our present study ALDH1L2 knockdown attenuated GSH production and induced PAI-1 in kidney cells (Fig. 7A, 7B, 7C).

In our study, inhibition of mir-219a-5p mainly suppressed fibronectin expression, and its effect on other ECM proteins, such as collagens, was marginal (Fig. 3, Supplementary Fig. 6). Even though plasmin mainly mediates fibronectin degradation and remodeling^4^, global knockout of PAI-1 attenuated both fibronectin and collagen deposition in kidney fibrosis^37^. In addition, PAI-1 inhibitors including TM5441 reduced overall ECM accumulation in diabetic nephropathy^38^. There are a few possibilities that may explain why mir-219a-5p did not have significant effects on collagen deposition in our study. First, according to the histological examinations of mir-219a-5p and ALDH1L2 (Fig. 1E, Fig. 5C), mir-219a-5p inhibition to regulate ALDH1L2 and fatty acid oxidation is mainly localized in renal tubules. In kidney, because of the high energy requirement, proximal tubule is the major renal compartment using fatty acid as a fuel source^39^. Therefore, the effect of mir-219a-5p inhibition in ECM remodeling is relatively limited in renal proximal tubules. However, global PAI-1 deficiency or PAI-1 inhibitor treatment can function on all renal cells, such as myofibroblasts and macrophages, to regulate ECM deposition. In fact, both PAI-1 knockout and inhibitor treatment were shown to suppress the inflammation as well as the transcription of ECM proteins in kidney^37,38^. Second, mir-219a-5p may have multiple potential target genes according to our microRNA target prediction and the Ago2-IP/RNA-seq result (Fig. 4). Although we confirmed ALDH1L2 as a direct target of mir-219a-5p in renal fibrosis, other target genes of mir-219a-5p may modulate ECM remodeling and collagen deposition as well. Finally, we only examined renal fibrosis up to two weeks after UUO injury. It is unclear whether the changes in fibronectin may affect collagen deposition at later time-points or in the long term.

In conclusion, this study has demonstrated the regulation of mir-219a-2 by DNA methylation in renal fibrosis, unveiling a novel crosstalk between epigenetic mechanisms. Specifically, fibrotic stress leads to hypermethylation of the mir-219a-2 gene, leading to the down-regulation of mir-219a-5p and a consequent increase in its target ALDH1L2 expression. Upon expression, ALDH1L2 promotes GSH to suppress PAI-1 expression, resulting in fibronectin degradation and less renal fibrosis.

## Methods

### Animal models

C57BL/6J mice were originally from The Jackson Laboratory (Bar Harbor, ME), bred and housed in Charlie Norwood VA Medical Center animal facility. Male mice of 8–12 weeks old were used for bilateral kidney ischemia surgery or unilateral ureter obstruction (UUO) surgery. All animal experiments followed the protocol approved by the Institutional Animal Care and Use Committee in Charlie Norwood VA Medical Center.

Bilateral kidney ischemia-reperfusion was conducted as described before^40^. Briefly, mice were anesthetized with 60mg/kg pentobarbital and kept on a homoeothermic blanket to maintain the body temperature at 36.5°C. Both kidney pedicles were clamped with micro-aneurysm clips for 25 minutes to induce kidney ischemia. The clips were released for kidney reperfusion and the mice were kept for 1 week or 1 month to collect kidneys. Sham operation was performed without renal pedicle clamping.

UUO was induced in mice as previously^41^. Briefly, mice were anesthetized with 60mg/kg pentobarbital and kept on a homoeothermic blanket for body temperature maintenance. The left ureter was ligated at two points with 4 − 0 silk suture to block urine and a cut was made between these two ligations. Sham operation was performed without ureter ligation and cut for comparison in in situ hybridization experiment. The contralateral kidneys were used in other experiments as the control for comparison purposes.

### Genome-wide DNA methylation sequencing

The genomic DNA samples were extracted from mouse kidney cortex and outer medulla with QIAmp DNA Blood Mini kit from Qiagen (Germantown, MD) according to the manufacturer’s manual. The Reduced Representative bisulfite sequencing and reads alignment were conducted in Cancer Center Genomic Core Facility at Augusta University as described before^42^.

### Pyrosequencing

The differentially methylated regions associated with the mir-219a-2 gene from genomic sequencing analysis were examined by pyrosequencing conducted by EpigenDx, Inc.(Hopkinton, MA) to conflrm the methylation levels. The sequencing information was shown in Supplementary Table 2.

### RNA Extraction

Total RNA from kidneys or cultured cells was extracted with miVana miRNA Isolation kit (Thermo Fisher Scientific, Carlsbad, CA) or GeneJet RNA purification kit (Thermo Fisher Scientific, Carlsbad, CA) following the manufacturer’s instructions. Kidneys were ground in liquid nitrogen and immediately lysed in a lysis buffer. Cells were lysed with lysis buffer in the culture dishes.

### Reverse transcription and Quantitative real-time PCR (RT and qPCR)

To quantify mRNA expression, 1μg of total RNAs were reversely transcribed into cDNA with iScript cDNA synthesis kit (Bio-Rad, Hercules, CA). qPCR was performed using iTaq Universal SYBR Green Supermix (Bio-Rad, Hercules, CA) with 18s rRNA as internal normalization control and the quantification was done using ΔCt values. The primers were synthesized by Integrated DNA Technologies (Coralville, IA), and the sequence was shown in Supplementary Table 3. To examine microRNA expression, 40 nanograms of total RNAs were reversely transcribed using TaqMan^®^ MicroRNA Reverse Transcription Kit (Thermo Fisher Scientific, Carlsbad, CA) and qPCR was performed using TaqMan^™^ Universal PCR Master Mix (Thermo Fisher Scientific, Carlsbad, CA) with detecting primers from TaqMan^®^ MicroRNA Assays (Thermo Fisher Scientific, Carlsbad, CA). Small nuclear RNA 202 was used as internal normalization control and the quantification was done using ΔCt values. All qPCR was performed by The Applied Biosystems^®^ 7500 Real-Time PCR System (Thermo Fisher Scientific, Carlsbad, CA)

### In situ hybridization

Kidney samples were fixed in 4% paraformaldehyde overnight at 4°C and balanced in 20% sucrose in PBS. Fresh cryo-sections of 7μm were air-dried for 10 minutes and briefly fixed in 4% paraformaldehyde for 20 minutes. After PBS wash, the slides were treated with proteinase K (0.5μg/ml) for 10 minutes at room temperature and rinsed with PBS. The hybridization was then performed with IsHyb in situ hybridization kit (Biochain Institute Inc., Newark, CA) following the manufacturer’s instructions. Briefly, the slides were incubated with a pre-hybridization solution at 63°C for 3hrs. The hybridization mixture with 0.5 nM DIG-labelled locked nucleic acid (LNA) probe (Exiqon, Germantown, MD) in 100ul hybridization solution was heated at 65°C for 5 min to linearize probes and then chill on ice. The slides were incubated with a hybridization mixture at 58°C overnight. After the SSC solution washes and blocking, the slides were exposed to anti-DIG alkaline phosphatase-conjugated antibody. Finally, the signal was developed with NBT/BCIP solution.

### In vitro flbrosis models

The following renal cells were used for in vitro treatment to induce fibrosis: (1) The mouse proximal tubular cell (BUMPT) line was originally obtained from W Lieberthal and JH Shwartz at Boston University and maintained in DMEM culture medium with 10% FBS^43^. BUMPT cells were stably transfected with empty vectors (pCMV-MIR, OriGene, Rockville, MD) or mir-219a-2 overexpression plasmids (OriGene, Rockville, MD) to examine the role of mir-219a-5p in renal fibrosis. (2) rat proximal tubular cell (RPTC) was originally from Dr. Ulrich Hopfer^44^. RPTC cells were maintained in DMEM/F12 medium with 10% FBS and transiently transfected with miRIDIAN miRNA mimics negative control #1 or miRIDIAN mir-219a-5p mimics (Dharmacon, Lafayette, CO) to examine the role of mir-219a-5p. (3) NRK-49F cell line was a renal fibroblast cell line purchased from ATCC (Manassas, VA). NRK-49F cells were maintained in DMEM with 10% FBS and transiently transfected with negative control or mir-219a-5p mimics to examine the role of mir-219a-5p. Cells were treated at 24 hours after transfection to induce fibrosis. (4) HEK293 cell line was from ATCC (Manassas, VA) and maintained in MEM Medium with 10% FBS. The cells were transiently transfected with negative control vs mir-219a-5p mimics, or ON-TARGETplus Non-targeting siRNAs negative control pool vs aldehyde dehydrogenase 1 family member L2 (ALDH1L2) siRNA (targeting sequence: AGAAAGAGCCACUCGGUGU) (Horizon, Lafayette, CO) to examine the role of mir-219a-5p or ALDH1L2. TM5441 (MedChemExpress, Monmouth Junction, NJ) was used to inhibit PAI-1. (5) Human kidney proximal tubular cell line (HK2) was from ATCC (Manassas, VA) and maintained in DMEM/F12 medium with 10% FBS. The cells were transiently transfected with negative control or mir-219a-5p mimics.

For hypoxia-induced fibrosis, the cells were incubated in a full culture medium in hypoxia (1% O_2_) for 24–72 hours to induce fibrosis. Cells cultured in normoxia were used for comparison. To induce fibrosis by TGF-β1 (EMD Millipore, Burlington, MA), cells were cultured in a serum-free medium with 10 or 20 ng/ml TGF-β1 for 24–72 hours. Cells without TGF-β1 treatment were used for comparison.

### Immunoblot analysis

Cultured cells were lysed in 1XSDS buffer (72.5mM Tris-HCl, pH 6.8, 2% SDS, 10% glycerol) with proteinase inhibitor cocktail (Sigma-Aldrich, St. Louis, MO) and benzonase (EMD Millipore, Burlington, MA) supplement for immunoblots. Kidney tissue was homogenized in 1XSDS buffer to obtain lysates for immunoblots. The lysates were separated by 10% SDS-PAGE gels and transferred to PVDF membrane. After blocking with 5% milk in TBS with 0.05% Tween 20, the membranes were incubated with specific primary antibodies [anti-fibronectin (ab2413, Abcam, Waltham, MA); anti-α-smooth muscle actin (SMA) (Abcam, ab5694); anti-col IV (Abcam, ab6586); anti-Calnexin (ThermoFisher, PA5–34757); anti-ALDH1L2 (gift from Dr. Sergey Krupenko in University of North Carolina, Chapel Hill, NC); anti-ALDH1L2 (21391–1-AP, Proteintech, Rosemont, IL); anti-GAPDH (Cell Signaling, #2118); anti-cyclophilin b (Cell signaling, #43603)] diluted in 5% milk at 4°C overnight. The signal was detected by HRP-labelled secondary antibodies (ThermoFisher, Madison, WI) followed by enhanced luminol-based chemiluminescent substrate exposure (Bio-rad, Hercules, CA). The signal was recorded by MyECL Imager (ThermoFisher, Madison, WI) or KwikQuant imager (Kindle Biosciences, LLC, US), or X-ray film exposure. Image J 1.53e (http://imagej.nih.gov/ij) was used for densitometry calculation and the densitometry value of target protein was normalized by internal loading control for comparison purposes.

### In vivo LNA delivery

LNAs of negative control sequence or anti-mir-219a-5p (Exiqon/Qiagen, Germantown, MD) were injected into mice through the tail vein. LNAs were dissolved in nuclease-free PBS at a concentration of 5mg/ml. Two injections of 20 mg/kg of LNAs were delivered two days before UUO surgery and three days after UUO surgery respectively. Animals were sacrificed at 2 weeks after UUO surgery. In this experiment, the contralateral kidneys were used as a control for comparison with kidneys with ureter obstruction.

### Immunohistochemical staining and Masson’s Trichrome staining

Mouse kidneys were harvested at the end of experiments and fixed in 4% paraformaldehyde overnight. The kidneys were dehydrated and embedded in paraffin. Cross sections of 5μm were used for immunohistochemical staining and Masson Trichrome staining.

For immunohistochemical staining, kidney sections were deparaffined and rehydrated. The sections were steamed for one hour in 10 mM sodium citrate (pH 6.0) or 1 mM EDTA (pH 8.0) with 0.05% Tween 20 for antigen retrieval and incubation in hydrogen peroxide to inhibit endogenous peroxidase. Then the sections were blocked in a blocking buffer containing 2% BSA, 0.2% non-fat milk, 0.8% Triton X-100 in PBS and incubated with primary antibody [Anti-Fibronectin (Abcam, ab2413); Anti-α-SMA (Agilent, M085129–2); Anti-ALDH1L2 (gift from Dr. Sergey Krupenko); Anti-PAI-1 (ThermoFisher, PA5–115715)]. diluted in blocking buffer at 4°C overnight. HRP-polymer labeled secondary antibodies (Vector Laboratories, Burlingame, CA) were used to amplify the signal. Finally, the signal was developed by a DAB kit from Vector Laboratories (Burlingame, CA). For the fibronectin staining experiment, the nuclei were counterstained by hematoxylin. For quantification of PAI-1 signaling, 10–20 images (200X) were randomly taken in the cortical area of each specimen. The tubules with significant induction of PAI-1 were selected and the percentage of the tubular area was analyzed by Image J 1.53e (http://imagej.nih.gov/ij).

The Trichrome Stain (Masson) Kit from Sigma-Aldrich (St. Louis, MO) was used for Masson’s Trichrome staining. The kidney sections were stained following the manufacturer’s instructions and examined by bright field microscopy. Minimally 30 images were taken to cover the whole cortical and outer medulla area for collagen deposition analysis. Image J 1.53e was used to calculate the percentage of area with a positive collagen signal.

### RNA-induced silencing complex immunoprecipitation (RISC-IP) and RNA-Sequencing

HEK293 cells were co-transfected with Flag-Ago2 overexpression plasmids (Addgene, Watertown, MA)^45^ and 100nM RNA oligos from Dharmacon (Lafayette, CO) (Negative control vs mir-219 mimics). The cells were lysed with lysis Buffer [150 mM KCl, 25 mM Tris-HCl, pH 7.4. 5mM EDTA, 0.5% NP-40, 5 mM DTT] containing proteinase cocktail from Sigma-Aldrich (St. Louis, MO) and 100 U/ml SUPERase•In^™^ RNase Inhibitor (Thermo Fisher Scientific, Carlsbad, CA). RISC complexes were pulled down by anti-Flag-agarose (Sigma-Aldrich, St. Louis, MO) and the RNAs in the RISC complex were extracted with miVana miRNA Isolation kit for RNA sequencing. The high-through mRNA deep sequencing with a minimum of 6G raw data/sample and the standard quantification data analysis was performed by Novogene (Durham, NC).

### microRNA target Luciferase assay

HEK293 cells were co-transfected with luciferase DNA plasmids and 100nM RNA oligos from Dharmacon. Three DNA plasmids were used for comparison: Empty Vector (pMIR-REPORT Luciferase from Thermo Fisher Scientific, Carlsbad, CA), ALDH1L2 (pMir-REPORT Luciferase plasmid inserted with predicted mir-219a-5p binding site in 3’UTR of human ALDH1L2), and Mutated (pMIR-REPORT luciferase plasmid inserted with mutated mir-219a-5p binding site) (Supplementary Fig. 9). At 24 hours after transfection, the cell lysates were collected with reporter lysis buffer (Promega, Madison, WI) and the luciferase activity was examined with Luciferase Assay System (Promega, Madison, WI) using a Tecan plate reader.

### GSH Measurement

Cells were plated into a 96-well plate and GSH level was measured with GSH-Glo Glutathione Assay from Promega (Madison, WI) following the assay manual.

### Statistics.

Data were expressed as mean ± SD and analyzed with Microsoft Excel or GraphPad Prism 9. Student’s t-test was used to show the significant difference between two groups. One-way ANOVA analysis with multiple comparisons specified in Figure Legends was used for multigroup difference analysis. A p-value less than 0.05 was considered statistically significant.

## Data Availability

All the data supporting the finding of this article are available upon request to the corresponding authors.
